# Comparison of Atorvastatin and Rosuvastatin in Reduction of Inflammatory Biomarkers in Patients with Acute Coronary Syndrome

**DOI:** 10.7759/cureus.4898

**Published:** 2019-06-14

**Authors:** Besham Kumar, Mir Ali Asghar Shah, Rajesh Kumar, Jai Kumar, Aurangzeb Memon

**Affiliations:** 1 Internal Medicine, Jinnah Postgraduate Medical Center, Karachi, PAK; 2 Internal Medicine, Liaquat University of Medical and Health Sciences, Hyderabad, PAK; 3 Internal Medicine, Liaquat University of Medical and Health Sciences, Jamshoro, PAK

**Keywords:** hmg-coa reductase inhibitors, atorvastatin, rosuvastatin, high sensitivity c-reactive protein, crp, acute coronary, inflammatory biomarkers, open label trial

## Abstract

Introduction

High-sensitivity C-reactive protein (hs-CRP) has emerged to be a very useful and reliable clinical marker of primary as well as secondary cardiovascular morbidity and mortality. Elevated hs-CRP contributes to underlying atherogenesis and worsens disease prognosis. Along with their lipid-lowering properties, statins also contribute to the alleviation of micro-inflammation and reduces pro-inflammatory markers. The aim of this study is to compare the effects of rosuvastatin and atorvastatin in lowering hs-CRP levels in statin-naive patients admitted with acute coronary syndrome (ACS).

Methods

In this prospective, open-label randomized trial, group A was given rosuvastatin 40 mg daily and group B was given atorvastatin 20 mg daily along with standard post-ACS therapy. Lipid profile (mg/dL), hs-CRP (mg/L) and erythrocyte sedimentation rate (ESR) (mm/Hr) were recorded and measured as the baseline (before starting therapy) and then again after four weeks. The data were analyzed using SPSS for Windows version 22.0 (IBM Corp., Armonk, NY).

Results

With four weeks of treatment, both group A and B showed statistically significant reduction in serum hs-CRP levels (p<0.0001). In group A, there was a mean 51% decrease in hs-CRP levels, and in group B, a 35% reduction was seen. Group A showed markedly low hs-CRP levels than group B after four weeks of therapy (18.46 ± 6.35 vs. 24.67 ± 8.45) (p<0.0001). Group A showed mean 16% decrease in ESR levels as compared to 14% decrease in group B. Group A showed lower ESR levels than group B after four weeks of therapy (19.59 ± 11.83 vs. 20.52 ± 12.13) (p<0.0001).

Conclusion

Rosuvastatin showed a 50% decrease and atorvastatin showed a 35% reduction in serum hs-CRP levels in statin-naive ACS patients. Rosuvastatin has a more effective role in reducing micro-inflammation in ACS patients.

## Introduction

Over the past few years, high-sensitivity C-reactive protein (hs-CRP) has emerged out to be a very useful and reliable clinical marker of cardiovascular (CV) risk. It is an acute phase reactant and has been predicting both primary and secondary risks of a CV event. It has also been suggested in the research that elevated levels of inflammatory biomarkers - hs-CRP and erythrocyte sedimentation rate (ESR) - point towards subclinical atherosclerosis long before a major acute coronary event occurs [1-2].

In a recent meta-analysis, including patients with acute coronary syndrome (ACS), it was deduced that patients with moderately elevated hs-CRP (3.1-10 mg/dL) and severely elevated hs-CRP (>10mg/dL) have 1.40 times and 2.18 times greater long-term risk of recurrent CV events or even death respectively [3]. Hs-CRP has not only been regarded as a predictor of adverse cardiovascular outcomes and atherosclerosis but also as a mediator. Hs-CRP has been established to play a critical role in all steps of atherogenesis such as complement activation, activity of macrophages, inflammatory cytokine release, tissue factor induction, endothelial dysfunction, and production of nitric oxide [4].

The largest trial conducted to study the effect of statins on hs-CRP levels was the JUPITER study (Justification for the Use of Statins in Prevention: an Intervention Trial Evaluating Rosuvastatin). With 20 mg rosuvastatin, hs-CRP levels were reduced by 37%. Reduction in hs-CRP levels significantly reduced the risk of all primary major cardiovascular events including myocardial infarction (MI), stroke, arterial revascularization, hospitalization for unstable angina (UA), or death from cardiovascular causes [5]. Since then, research has been focused on the role of statins and other agents in reducing hs-CRP levels [6].

Statins impart pleiotropic effects - that is, they are simultaneously capable of producing more than one benefit, and in cardiovascular risk reduction, they accomplish more than simply lowering cholesterol on the vasculature which contributes to their anti-anginal and anti-ischemic traits. Statins stop the inflammatory process in the endothelium of vessel walls through molecular pathways, involving both innate and adaptive immune systems. Statins inhibit the mevalonate pathway and isoprenoid formation, resulting in increased availability of nitric oxide and protection from ischemia-reperfusion injury. Statins also improve endothelial function, enhance ischemic vasodilatory response, and modulate inflammation [7-8]. Rosuvastatin and atorvastatin are both high-intensity statins and have shown great results in lowering low-density lipoprotein (LDL) which is primarily responsible for all adverse outcomes of hyperlipidemia [9]. Randomized trials, as well as meta-analyses, have shown superior effects of rosuvastatin in reverting coronary atherosclerotic plaques and reduction of hs-CRP levels [10-11]. However, to the best of our knowledge, no such comparative trial has been published from Pakistan to discuss the superior statin in the South Asian population. The aim of this study is to compare the effects of rosuvastatin and atorvastatin in lowering hs-CRP levels in statin-naive patients admitted with acute coronary syndrome.

## Materials and methods

We conducted a prospective, open-label, randomized trial in the cardiovascular unit of a tertiary care hospital in Pakistan from January till December 2018. We took consent from all patients, and the study was approved by the ethical review committee of the institute. In order to compare the effects of atorvastatin and rosuvastatin on the inflammatory markers in patients of ACS, we included adult patients of age 18 years and above, of both genders, diagnosed with STEMI, NSTEMI, or UA according to World Health Organization criteria [12] who were not taking statins.

We excluded patients who were taking either statins and/or any other drug which lower serum lipid levels, patients with a history of statin hypersensitivity, patients in whom statins were contraindicated, patients with severe cardiac dysfunction (ejection fraction < 30%), and any other comorbidity including severe anemia and hepatic or renal failure. Patients who were surgically managed were also excluded. We also excluded pregnant or lactating women.

After fulfilling the inclusion criteria and signing the informed consent, the patients were randomized into two groups. Group A received 40 mg rosuvastatin daily and group B received 20 mg atorvastatin daily along with their standard regime which included aspirin, clopidogrel, beta-blocker, nitrates, and an angiotensin-converting enzyme inhibitor. Serum lipid profile, hs-CRP, and ESR were recorded for all patients at baseline (before starting therapy) and then again after four weeks. Lipid profile was measured using Vitros 250 automatic analyzer (Ortho Clinical Diagnostics, Raritan, NJ). Hs-CRP levels were measured using Turbox hs-CRP kit (for protein analyzer Turbox plus) by turbidimetry method. ESR was measured using Westergren method.

Data was entered and analyzed using the IBM SPSS Statistics for Windows, Version 22 (IBM Corp., Armonk, NY). Mean and standard deviation were calculated for continuous variables including lipid profile, hs-CRP, and ESR levels for groups A and B. We correlated the means within each group (baseline vs. four weeks) by applying dependent T-test and within the two groups (at four weeks of group A vs. group B) by applying independent T-test. P value ≤0.05 was taken as significant.

## Results

At baseline there were 104 patients in group A and 103 in group B. By four weeks, there were 99 patients in group A and 94 in group B. The rest were lost to follow-up.

The changes in hs-CRP levels in both groups during the study period is shown below in Table 1.

**Table 1:** Mean change in hs-CRP levels (mg/L) during the study period Group A: Rosuvastatin 40 mg daily Group B: Atorvastatin 20 mg daily * In each group compared at four weeks from baseline ** Inter-group comparison at four weeks

Groups	At Baseline	At Four Weeks	Mean change (%)	P-value*	P-value**
Group A	38.34 ± 10.23	18.46 ± 6.35	51.85 ± 3.79	<0.0001	<0.0001
Group B	38.19 ± 12.38	24.67 ± 8.45	35.40 ± 3.17	<0.0001	

With four weeks of treatment, both rosuvastatin and atorvastatin showed a statistically significant reduction in serum hs-CRP levels (p<0.0001). In the rosuvastatin group, there was a mean 51% decrease in serum hs-CRP levels; in the atorvastatin group, there was a mean 35% decrease. In inter-group comparison, the rosuvastatin group showed markedly low serum hs-CRP levels than the atorvastatin group after four weeks of therapy (18.46 ± 6.35 vs. 24.67 ± 8.45) (p<0.0001).

The changes in ESR levels in both groups during the study period is shown below in Table 2.

**Table 2:** Mean change in ESR levels (mm/Hr) during the study period Group A: Rosuvastatin 40 mg daily Group B: Atorvastatin 20 mg daily * In each group compared at four weeks from baseline ** Inter-group comparison at four weeks

Groups	At Baseline	At Four Weeks	Mean change (%)	P-value*	P-value**
Group A	23.47 ± 13.53	19.59 ± 11.83	16.53 ± 1.25	0.031	<0.0001
Group B	23.88 ± 11.09	20.52 ± 12.13	14.07 ± 0.93	0.043	

With four weeks of treatment, both rosuvastatin 40 mg group and atorvastatin 20 mg group showed a statistically significant reduction in ESR levels (p <0.05). In the rosuvastatin 20 mg group, there was a mean 16% decrease in ESR levels and in the atorvastatin 40 mg group, there was a mean 14% decrease. In inter-group comparison, the rosuvastatin 20 mg group showed lower ESR levels than the atorvastatin 40 mg group after four weeks of therapy (19.59 ± 11.83 vs. 20.52 ± 12.13). The difference was statistically significant (p<0.0001).

The mean change in lipid profile in both groups during the study period is shown below in Table 3.

**Table 3:** Mean change in lipid profile (mg/dL) during the study period Abbreviations: TC, total cholesterol; HDL, high-density cholesterol; LDL, low-density cholesterol; VLDL, very low-density cholesterol; TG, triglycerides. Group A: Rosuvastatin 40 mg daily Group B: Atorvastatin 20 mg daily * In each group compared at four weeks from baseline ** Inter-group comparison at four weeks

Groups	Lipid Profile	At Baseline	At Four Weeks	Mean change (%)	P-value*	P-value**
Group A	TC	223.43 ± 33.56	149.56 ± 26.78	73.87 ± 6.78	<0.0001	0.301
	HDL	38.78 ± 9.48	41.02 ± 9.56	5.77 ± 0.08	0.09	0.523
	LDL	198.36 ± 53.66	66.11 ± 21.84	66.67 ± 5.92	<0.0001	0.270
	VLDL	51.11 ± 29.38	34.16 ± 16.23	33.16 ± 4.47	<0.0001	0.01
	TG	143.60 ± 53.39	129.23 ± 46.74	10.00 ± 1.24	0.04	0.02
Group B	TC	220.91 ± 35.84	153.56 ± 26.78	30.48 ± 2.52	<0.0001	
	HDL	39.28 ± 10.54	40.17 ± 8.87	2.21 ± 1.58	0.524	
	LDL	200.49 ± 54.14	70.87 ± 36.57	64.65 ± 3.24	<0.0001	
	VLDL	50.56 ± 28.53	39.27 ± 12.34	22.32 ± 5.67	0.0005	
	TG	145.07 ± 48.81	115.43 ± 36.98	20.43 ± 2.42	<0.0001	

As seen in Table 3, in both rosuvastatin and atorvastatin groups, a mean favorable change was observed in total cholesterol (TC), low density lipoprotein (LDL), very low density lipoprotein (VLDL), and triglycerides (TGs) that was statistically significant over a period of four weeks (p<0.05). When inter-group comparison was done, only the change in mean VLDL and TGs was statistically significant (p<0.05).

## Discussion

This study has evaluated that although the lipid-lowering effects of atorvastatin and rosuvastatin are comparable, the latter has a more profound impact on the reduction of pro-inflammatory markers, especially hs-CRP, which is an established predictor of cardiovascular morbidity and mortality. When tested between the groups, rosuvastatin showed significantly lower hs-CRP levels than atorvastatin at the end of the study.

To the best of our knowledge, this is the first published data on the comparison of anti-inflammatory effects of atorvastatin and rosuvastatin from Pakistan. However, this trial was open-label and conducted in only one center which makes its methodology not very robust. Although there has been a reduction in hs-CRP levels at the end of the study, the levels were still higher than the upper normal limit. This deduces that the patients should’ve followed the treatment for a longer duration to reach the safe limits of hs-CRP. The case with ESR is similar. However, the study couldn’t be stretched for a longer duration.

The literature regarding the superiority of either statin in the reduction of pro-inflammatory markers is not concrete. In a randomized open-label trial with diabetic patients, only atorvastatin significantly reduced hs-CRP levels (p=0.02) while rosuvastatin did not. There was also no statistically significant difference between the groups [13]. In another randomized double-blind trial, there was a statistically significant reduction in hs-CRP levels with both atorvastatin and rosuvastatin. At 16 weeks, there was a 40% reduction with rosuvastatin and 34% reduction with atorvastatin. However, the differences were not statistically significant between the groups [14]. Although JUPITER trial has been a landmark trial in establishing the role of rosuvastatin in protective anti-inflammatory effects against CV risk, it did not compare the two statins or rosuvastatin with any other lipid-lowering agent [5]. In another meta-analysis, rosuvastatin displayed a more pronounced effect in reducing LDL than atorvastatin (P < 0.001), but not in increasing HDL (P = 0.22) and reducing hs-CRP (P = 0.68) [10]. In another study with obese type 2 diabetic patients, both atorvastatin and rosuvastatin significantly reduced hs-CRP levels; however, the differences were not significant between the groups [15].

Khurana et al. have been substantial in supporting the role of rosuvastatin against inflammatory markers. It was an open-label trial which showed that hs-CRP levels were significantly decreased after four weeks in both rosuvastatin and atorvastatin groups (P < 0.001). Between the groups, the test revealed that the rosuvastatin group showed significantly lower hs-CRP levels as compared to the atorvastatin group (P< 0.05). The mean percentage decrease in hs-CRP after four weeks in the rosuvastatin group was 44% and that in atorvastatin group was 35% [16]. In another recent study with ACS patients, both atorvastatin 80 mg and rosuvastatin 40 mg were effective in causing a statistically significant reduction in hs-CRP levels. Rosuvastatin was more effective in reducing hs-CRP levels than atorvastatin [17].

Rosuvastatin was also studied as pre-treatment before percutaneous coronary intervention (PCI) following myocardial infarction. After a mean follow-up of 12 months, a major cardiovascular event (MACE) occurred in 20% controls (not receiving rosuvastatin) as compared to 9% in the rosuvastatin group (P< 0.05). Hs-CRP levels were less elevated in the rosuvastatin group than in the control group at 24 hours after PCI. Rosuvastatin loading was an independent predictor of a reduction in the risk of MACEs at 12 months [18].

Elevated hs-CRP has been established as a prognostic indicator of new MACE and mortality in patients with ACS [19]. Effective medical intervention to control this underlying micro-inflammation has a critical role in preventing mortality and bettering disease outcome. Statins have shown striking results in reducing hs-CRP levels in patients with ACS. While the choice of statin has been controversial with some literature supporting atorvastatin and other supporting rosuvastatin; the role of rosuvastatin has been more beneficial.

Longitudinal double-blind randomized trials are recommended to follow ACS patients until hs-CRP levels return to normal, and to assess the impact of statin choice in all-cause mortality, cardiac mortality, and new MACEs.

## Conclusions

In statin-naïve patients admitted with acute coronary syndrome, the role of atorvastatin and rosuvastatin has been comparable in optimizing the lipid profile. Both groups showed a statistically significant reduction in ESR and hs-CRP levels at four weeks within groups as well as between groups. Rosuvastatin showed a 50% decrease in serum hs-CRP levels and atorvastatin showed a 35% reduction. Rosuvastatin has a more effective role in reducing micro-inflammation in ACS patients which helps improve the disease outcome.
